# Investigating factors influencing fatalities and injuries in animal-vehicle crashes using a random parameters logit model and ensemble machine learning approaches

**DOI:** 10.1371/journal.pone.0331197

**Published:** 2025-09-02

**Authors:** Md Nabil Zawad, Mohammed Almannaa, Khalid F. Alkahtani

**Affiliations:** Department of Civil Engineering, College of Engineering, King Saud University, Riyadh, Saudi Arabia; Tongji University, CHINA

## Abstract

Animal-vehicle crashes (AVC) pose risks in rural areas, often leading to casualties and injuries. Despite their infrequent occurrence, AVC can have significant consequences, especially when larger animals are involved. This study investigates factors contributing to fatalities and injuries resulting from animal-involved collisions. It examines 24 variables using 1403 animal-vehicle crash observations on intercity and major intra-city roads from 2016–2021. The study employs a random parameters logit model (RPLM) and ensemble machine learning approaches to explore the contributory factors in crashes. The RPLM accounts for unobserved heterogeneity, identifying significant variables. Meanwhile, the ensemble learner and Shapley Additive exPlanations (SHAP) provide further insights. Key findings show that expressways, roads with one or two lanes per direction, horizontal curvature, and structurally poor pavement surfaces increase the risk of severe crashes, i.e., fatalities and injuries. Side fence barriers and speed bumps also impact crash severity. The absence of side fencing and damaged fencing both positively influence severe crashes, while the presence of speed bumps is likely to increase severe crashes. Camel exposure, vacation-period crashes, and adverse weather also play positive roles. However, heavy truck involvement is negatively associated with severe crashes. Policymakers and road safety authorities can use these findings to implement effective countermeasures to prevent such collisions.

## Introduction

### Background

Animal-vehicle crashes (AVC) pose a significant road safety concern globally, particularly in rural regions. These incidents result in severe human injuries, fatalities, property damage, and wildlife loss. Despite a generally low human fatality count, AVC demand attention due to recent crash trends, associated costs, and severe patient aftermath [1]. In 2021, the USA reported over 280,000 AVC, leading to more than 27,000 injuries and 156 fatalities [2]. In Canada, the Insurance Corporation of British Columbia recorded an annual average of over 11,000 AVC from 2018–2022, including 900 injuries and 3 fatalities [3]. In Australia, Australian Associated Motor Insurers Limited received over 17,000 vehicle insurance claims in 2022 involving crashes with animals. According to their report, one in every seven vehicles engaged with wildlife had to be junked off due to the severe damage [4].

Economically, AVC cost the USA over USD 8 billion annually [5], while São Paulo state in Brazil incurs over USD 25 million annually [6]. AVC often involve large animals like kangaroos in Australia, moose and deer in the USA, Canada, and parts of Europe, and camels in Middle Eastern countries. Crashes involving larger animals like moose or camels, which typically weigh over 500 kg and stand 6–8 ft. tall, can cause long-term injuries, especially in the head, face, and cervical spine [7,8] in addition to fatalities and significant vehicle damage.

In Saudi Arabia (KSA), AVC, particularly involving Arabian camels (both domesticated and feral), are prevalent, causing approximately 600 collisions annually with a human fatality rate of 0.25 per collision [9]. Camel owners often graze their camels near highways, leading to increased AVC due to inadequate roadside protection. To aggravate the situation, roadside fencing installed to protect the highways from animal infiltration is often cut to make a passage for camels and sheep and even for vehicles to travel to the other side of the locality [10]. Besides camels and sheep, feral donkeys are also seen to approach human habitations in rural areas, increasing the likelihood of AVC [11].

To minimize the loss of human capital and protect the animals in KSA, especially camels which hold great cultural significance, it is essential to investigate more about animal-related collisions. While mitigation measures like using warning signs of particular shape and size [9], developing animal detection system using GPS [12], and deep learning models [13] have been proposed in context of Saudi Arabia, other preventive investigations are yet to be explored. Concerning crash severity, researchers in KSA have explored individual victim classes such as drivers of varying nationalities [14] and ages [15] while investigating overall crash severity. However, no attention has been given to understanding the contributory factors affecting the severity of animal-vehicle crashes. To fill this gap, this study aims to analyze the severity outcomes of animal-involved crashes and identify the contributory factors influencing fatalities and injuries, based on 1,403 events recorded over four years in Saudi Arabia. The analysis employs both statistical and machine learning approaches, with a focus on severe outcomes over no-injury cases due to their greater impact on human life and public health. The ultimate goal is to provide insights that can assist policymakers in enhancing road safety and reducing severe injuries and fatalities.

### Literature review

Crash injury severity has been a long-standing topic in road safety research, analyzing the relationship between human injury severity levels and influencing factors using statistical methods [16], or machine learning (ML) techniques [17]. However, limited studies have focused on AVC severity. This scarcity is often attributed to the inherent challenges in acquiring detailed and high-quality data on animal-vehicle collisions. Despite challenges, recent methodological advances have addressed underreporting issues using copula regression models [18,19].

Savolainen and Ghosh [19] Savolainen and Ghosh [20] conducted an early study in Michigan, USA, using a multinomial logit model to investigate severe injuries in AVC. The study highlighted that younger drivers (≤ 25 years) and female drivers were more likely to experience severe injuries. Additionally, horizontal curvature and speed limits exceeding 90 kph were identified as contributing factors to crash severity, emphasizing the role of roadway geometry and speed in crash outcomes.

Building on this, Al-Bdairi, Behnood [21] applied a random parameters logit model (RPLM) with heterogeneity in means and variances to crash data of vehicle collisions with deer and elk from Washington, USA. Their findings revealed that crashes occurring during daylight, on freeways, roads with high-speed limits (≥ 90 kph) were associated with higher injury severity. Ahmed, Cohen [22] explored deer-vehicle collisions in Pennsylvania, USA, using a correlated random parameter ordered logit model. Their analysis distinguished between factors contributing to severe injuries and fatalities, and property-damage-only (PDO) crashes. Airbag deployment was found to significantly increase the likelihood of severe injuries and fatalities indicating high-impact collisions with animals, while unbelted passengers, nighttime crashes, and younger drivers (≤30 years) were linked to PDO outcomes.

Gharraie and Sacchi [23] expanded the scope by investigating wildlife-vehicle collisions in Saskatchewan, Canada, using a structural equation modeling approach. Their results demonstrated that non-intersection sites, divided roadways, poor pavement conditions, wet road surfaces, heavy vehicle involvement, and bad weather significantly influenced crash severity. Horizontal curvature was again confirmed as a critical factor contributing to severe injury outcomes, aligning with findings from earlier studies.

In recent years, ML has been a popular choice in AVC severity research due to some of its advantages over conventional statistical models. Unlike statistical methods, ML models, non-parametric models, to be specific, do not require any assumptions regarding the underlying probability distribution of the data and presumed relationships between the response variable and predictor variables [24]. Moghaddam, Balali [25] utilized supervised ML methods, specifically CatBoost classifiers, to study AVC in Tennessee, USA. Their research primarily identified significant variables like speed limits, crash timing, and alcohol involvement but lacked detailed interpretive insights. Rahman, Das [26] adopted an unsupervised ML method, association rules mining, to analyze injury severity in Louisiana, USA. They identified unlit dark conditions, involvement of light trucks, younger drivers (≤ 25 years), and speed limits above 100 kph as critical factors.

While these studies provide a foundational understanding of AVC severity, they primarily focus on specific geographic regions or animal types. Moreover, variables like roadway conditions, animal types, and temporal factors are explored in isolation rather than in combination, leaving room for more comprehensive analyses.

Although ML models excel in computational capability and predictive accuracy, they often lack interpretability, which is a critical requirement for policymakers developing effective countermeasures for crash prevention. To address this limitation, many crash-severity studies have adopted post-hoc interpretability techniques like Local Sensitivity Analysis, Partial Dependence Plots (PDP), Local Interpretable Model-agnostic Explanations (LIME), and Shapley Additive exPlanations (SHAP) [27]. However, the application of ML to capture unobserved heterogeneity in crash data remains underdeveloped compared to statistical approaches [28,29].

This gap highlights the need for a hybrid approach combining statistical and ML methods to use their complementary strengths. Statistical models like the RPLM offer insights into variable significance and unobserved heterogeneity, while ML methods enhance predictive accuracy and can analyze complex datasets. Employing a hybrid approach that combines these models is increasingly recognized as a promising research direction [30]. While numerous studies have adopted this approach through comparative analyses of the methods’ results in the past [15,31,32], recent studies have shifted the focus on integrating traditional statistical models, particularly heterogeneity models, with machine learning techniques to form a cohesive connection and derive comprehensive conclusions [33–38].

### Research aim and contribution

This study contributes empirically by including new variables related to the conditions of road safety items at crash sites, which were not previously examined in the context of AVC severity analysis. These variables, listed in Table 1, include observing the existence and conditions of road marking, cat eyes, concrete barriers, metal cable barriers, side fence barriers, traffic signs, overhead signs, speed bumps, cleanliness of the roadway, and road shoulders, presence of U-turns, and obstacles on the sides of the road. The RPLM is employed to quantify variable significance and capture potential unobserved heterogeneity. Using the statistically significant variables from RPLM, the study further explores variable impacts on crash severity outcomes by utilizing Ensemble Machine Learning classifier (EL) and SHAP dependency plots. This research is the first to integrate RPLM and EL methods to investigate factors contributing to AVC severity. EL method was chosen for its superior predictive accuracy over other ML models in classifying severity outcomes [17]. This work is also the first to examine camel-involved collisions, which accounts for 88% of the dataset records.

**Table 1:** Summary statistics of the AVC dataset.

Data Type: Categorical						
No.	Attribute	Feature Name	Level	Code	Frequency	Percentage
1	**Temporal & Environmental**	Weekend	No	1	1048	75%
			Yes	2	355	25%
2		Time	Daytime	1	524	37%
			Nighttime	2	879	63%
3		Weather	Clear	1	1380	98%
			Dust/ Foggy/ Rainy/ Other	2	23	2%
4	**Roadway Characteristics**	Road Type	Undivided	1	499	36%
			Divided	2	304	21%
			Freeway	3	600	43%
5		Lanes (per direction)	1	1	510	36%
			2	2	764	54%
			3	3	129	9%
6		Maximum Speed Limit (km/h)	80	1	30	2%
			90	2	21	2%
			100	3	461	33%
			110	4	496	35%
			120	5	395	28%
7		Road Geometry	Straight road	1	1096	78%
			Horizontal curve	2	136	10%
			Not available	3	171	12%
8		Road Surface Condition	Good	1	1077	77%
			Potholes/ Cracks/ Others	2	326	23%
9	**Crash characteristics**	Vacation	No	1	880	63%
			Yes	2	523	37%
10		Heavy Truck Involvement	No	1	1237	88%
			Yes	2	166	12%
11		Crash Severity Outcome **(Response Variable)**	Property Damage Only (PDO)	0	689	49%
			Severe (Injury & Fatality)	1	714	51%
12		Type of Animal	Camel	1	1236	88%
			Sheep	2	43	3%
			Donkey	3	69	5%
			Dog	4	19	1%
			Unspecified	5	36	3%
13	**Condition of road safety and maintenance items**	Road painting	Good	1	713	51%
			Replace	2	456	32%
			Not applicable	3	234	17%
14		Cat eyes	Good	1	910	65%
			Replace	2	364	26%
			Not applicable	3	129	9%
15		Concrete barrier	Good	1	772	55%
			Replace	2	63	4%
			Not applicable	3	568	41%
16		Metal cable barrier	Good	1	739	53%
			Replace	2	200	14%
			Not applicable	3	464	33%
17		Side fence barrier	Good	1	125	9%
			Replace	2	41	3%
			Not applicable	3	1163	83%
			Not available	4	74	5%
18		Traffic signs	Sufficient	1	756	54%
			Replace	2	186	13%
			Not enough	3	461	33%
19		Overhead signs	Good	1	179	13%
			Replace	2	9	1%
			Not applicable	3	1157	82%
			Not available	4	58	4%
20		Speed bumps	Good	1	182	13%
			Replace	2	24	2%
			Not applicable	3	856	61%
			Not available	4	341	24%
21		Presence of U-turns	No	1	1091	78%
			Yes	2	312	22%
22		Obstacles on the sides of the road	Obstacles with protection	1	1172	84%
			without protection	2	231	16%
23		Cleanliness of the roadway	Acceptable	1	1122	80%
			Not acceptable	2	281	20%
24		Road shoulders/ Emergency Lane	Acceptable	1	1156	82%
			Not acceptable	2	247	18%
**Data Type: Numeric**						
**No.**	**Attribute**	**Feature Name**	**Mean**	**SD**	**Minimum**	**Maximum**
25	**Spatial**	Length of Road (km)	119	68.8	1	560

## Materials and methods

### Data description

Two datasets were utilized from the Saudi Ministry of Transportation and Logistic Services (MTLS): Crash Records and Roadway Condition Records. While the observations in the crash dataset were recorded at the time of the incident, the second dataset consisting of roadway evaluation reports observing both safety and maintenance items was collected later by another survey team. Although there was a separate column for animal-vehicle collision records in the crash dataset, we also manually reviewed crash observation remarks to ensure that all possible AVC cases were captured.

**Fig 1:**
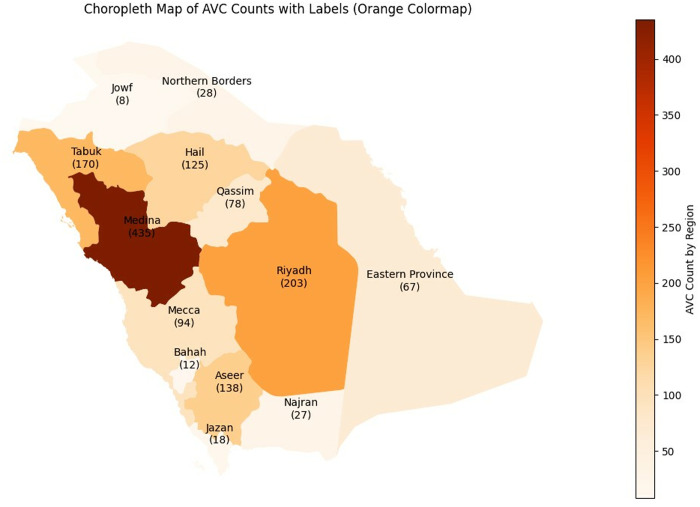
Animal-vehicle crash count per region (The shapefile is reprinted from SimpleMaps.com under a CC BY 4.0.).

From this process, 1,403 AVCs were identified and extracted from a larger dataset of 59,403 total crashes that occurred on intercity and major intra-city roads across all 13 provinces in Saudi Arabia over a period from October 2016 to February 2021. This subset represents approximately 2.3% of the total crash dataset and was included in its entirety, without any sampling or subjective selection criteria. Fig 1 illustrates the regional distribution of these animal-vehicle crashes. It is worth noting that Medina reported the highest number of (AVCs) in the dataset, followed by Riyadh.

The list of variables extracted from the two datasets with detailed descriptive statistics are shown in Table 1.

The dataset was modified for analysis, with two new variables: Weekend and Vacation, created from crash date records. AVC records on Fridays and Saturdays were labeled as Weekend, reflecting KSA’s official weekends. The Vacation variable indicates if crashes occurred during public holidays. Length of Road variable consists of the information about road length using the road ID number. This variable has been used as a proxy-variable for road classification to capture the effects of long-distance driving in animal-vehicle crash severity for each road type. The Road Geometry variable was added using crash location coordinates, but 171 data points were labeled ‘not available’ due to missing location information. The dataset includes information on animal types involved in crashes, obtained from crash observation notes. However, the animal’s name was not mentioned in the crash report for 36 AVC events. The distribution of crashes over time is grouped into two variables: daytime refers to the crashes that occurred between 6 AM and 6 PM, and nighttime includes the crash events that occurred at night. Any features not matching the roadway type or particular road section were labeled ‘not applicable’. The table also includes two distinct variables: Road Surface Condition, assessing pavement quality, and Cleanliness of the Roadway, checking for sand dunes or debris accumulation.

The crash severity outcome initially had three levels: fatality, injury, and property damage only (PDO). Due to fewer fatal outcomes (11%), they were combined with injury classes (40%) to avoid bias towards the majority class. This combination is labeled ‘Severe’ in subsequent sections.

## Methodology

**Fig 2:**
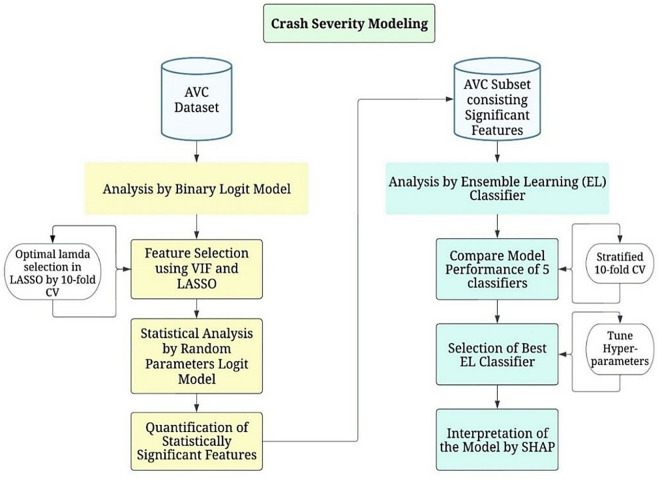
Methodological workflow of crash severity modeling.

The methodological framework of crash severity modelling is shown in Fig 2. As proposed, RPLM and EL classifiers were used in this study to explore the contributing factors in animal-involved road crash severity. RPLM contributed to finding statistically significant factors that affect the AVC severity outcome (either Severe or PDO). The identified significant variables in the RPLM were then investigated using the best EL classifier. Finally, the findings were explained with the help of SHAP summary and dependency plots.

It is worth noting that the methodological approach proposed here differs from conventional hybrid methods that employ machine learning for initial feature ranking before statistical interpretation. Instead, statistical methods (Binary Logit with VIF and LASSO, followed by RPLM) were prioritized for feature selection before applying the EL classifier with SHAP interpretation. This reverse sequence was chosen for three key reasons. First, to address potential multicollinearity and unobserved heterogeneity in our dataset through statistically rigorous feature selection; second, to ensure that selected features have statistical significance and theoretical relevance rather than merely predictive power; and third, to utilize ML’s pattern recognition capabilities on a refined feature set while exploring complex non-linear relationships and interaction effects through SHAP, which might not be fully captured by the RPLM alone.

### Feature selection

Redundant variables often cause multicollinearity among the independent variables which violates one of the general assumptions of logistic regression. Variance Inflation Factor (VIF) was used to detect the presence of such variable redundancy in the dataset. Although no strict values of VIF are set, a rule of thumb of 10 or 5 is often used [39]. In this study, a VIF value greater than 5 was set as the condition for dropping the highly correlated variables. An iterative approach was adopted instead of removing all highly correlated variables simultaneously. The initial results identified three variables with high VIF scores: Road Type (5.03), Side Fence (6.25), and Overhead Sign (6.22). Despite its lower score compared to the Side Fence variable, the Overhead Sign variable was removed first due to the greater significance of side fencing conditions in AVC compared to the overhead signs. After removal, remaining variables met the inclusion criteria. Post redundancy removal, the Least Absolute Shrinkage and Selection Operator (LASSO), an embedded method [40] was applied to prevent overfitting of the model. LASSO shrank 6 variables to zero, selecting 17 in total as potential predictors.

### Random parameters logit model (RPLM)

RPLM is a statistical model that improves reliability and prediction accuracy by accounting for unobserved variables. Adopting the mathematical background from the book of Washington, Karlaftis [41], the formulation of the model begins with the crash severity function which determines the AVC severity outcome. The basic form of it can be defined as:


$$y_{si}=\beta_{s}x_{si}+\varepsilon_{si}$$ (1)

where, *y*_*si*_ is the severity function which determines the probability of AVC severity outcome *s* in crash *i*. *β*_*s*_ is a vector of parameters to be estimated for AVC severity level *s*. These parameters represent the effects of various factors on crash severity. *x*_*si*_ is a vector of observed variables (predictors), and ***ε***_***si***_ is a random error term assumed as extreme values. The derivation of the random parameters logit model can be shown as:


$$\begin{array}{c} P_{i}(s)=\;\int \frac{EXP(\beta_{s}x_{si})}{\sum_{\forall \text{S}}EXP(\beta_{S}x_{Si})}\;f\left(\beta |\varphi \right)d\beta \end{array}$$ (2)

where *P*_*i*_*(s)* is the probability of the crash *i* that results in a discrete outcome *s* (*s* ∈ *S*), either Severe or PDO. In RPLM, some of the *β*s** are allowed to vary across individuals to capture unobserved heterogeneity. The weights used to determine *P*_*i*_*(s)* is governed by the *f*(*β*│*φ),* the density function of *β*. The function follows a specific distribution (normal/ log-normal/ triangular or others) which is described by the vector *φ*. If the estimated variance of the parameter distribution is statistically significant, it is confirmed to be a random parameter. The coefficients of RPLM were estimated using maximum simulated likelihood, with 500 Halton draws used for probability simulation, as suggested by Sarrias [42]. Several distributions for the random parameters were explored (such as, normal, log-normal, truncated normal, triangular, and uniform). The random parameters with normal distribution were found to yield better statistical fit than other form of distributions which aligns with the previous findings of crash severity analysis by Al-Bdairi, Behnood [21] Islam and Jones [43], and others. Statistical testing was carried out to determine the well-fitted model with the help of Akaike Information Criterion (AIC) and Bayesian Information Criterion (BIC). A likelihood ratio (LR) test was also conducted to compare the statistical significance of the RPLM model over a standard logit model (LM). The LR test statistic follows a chi-square distribution, with degrees of freedom equal to the difference in the number of parameters estimated in the models. The null hypothesis, that models with or without random parameters are equal, is rejected if the p-value is less than the chosen significance level. In this study, the LR test statistic based on the likelihoods of RPLM and LM was found as 6.26. The p-value of the chi-square test statistic with three degrees of freedom is 0.099, providing evidence at a 90% confidence interval in favor of the RPLM.

### Ensemble learning (EL) classifiers

EL classifiers combine multiple base machine learning algorithms. These base models are trained individually to predict crash severity outcomes, with results aggregated using a ‘majority vote’ method for categorical outcomes [44]. This study tested five ensemble machine learners—Random Forest [45], Extra Trees [46], CatBoost [47], XGBoost [48], and LightGBM [49] —using significant variables from the RPLM results. To avoid complexity, mathematical modeling of all these ensemble learners has not been elaborated upon in this subsection. Readers seeking a more detailed understanding of the algorithms are encouraged to refer to the citations provided alongside each ensemble learner.

The metrics used to evaluate and compare the prediction performance of the ensemble learners include Accuracy, Recall, Precision, Area Under the Curve (AUC), and F1-score. These metrics use the summarization of the prediction performance of a specific classifier and return a value between 0 and 1 based on the formulas. Table 2 shows a standard format of the confusion matrix, and the metrics formulas are shown in Equation (3–6).

**Table 2:** Confusion matrix.

		Predicted	
		Negative	Positive
**Actual**	**Negative**	True Negative (TN)	False Positive (FP)
	**Positive**	False Negative (FN)	True Positive (TP)


$$\text{Accuracy}=\frac{\text{TP}+\text{TN}}{\text{TP}+\text{FP}+\text{TN}+\text{FN}}$$ (3)

$$Recall=\frac{TP}{TP+FN}$$ (4)

$$Precision=\frac{TP}{TP+FP}$$ (5)

$$F1=2\times \frac{Recall\times Precision}{Recall+Precision}$$ (6)

In the confusion matrix, ‘positive’ implies the severe crash outcomes whereas ‘negative’ indicates crashes with no injuries, i.e., PDO. Using the matrix, Accuracy measures the proportion of correctly identified instances among all. It is considered a fair estimation of the classifier’s performance for class-balanced datasets. The unit of Accuracy ranges in [0, 1], where 1 represents a case with perfect prediction. Recall, also known as sensitivity or true positive rate (TPR), is an evaluator which is more concerned with the positive outcomes. It is expressed as the ratio between correctly identified positive instances and all positive instances. A classifier with higher sensitivity is recommended in case of crash severity modeling to avoid misjudgment and underestimation of the severity level. In addition to Accuracy and Recall, other evaluation metrics including Precision, AUC, and F1 Score provide better insights into the classifier’s performance. Precision evaluates how many of the crashes predicted as severe were actually severe. AUC quantifies the overall performance of the model. A model with an AUC close to 1 demonstrates strong discriminatory power, whereas an AUC near 0.5 indicates random guessing. F1 Score serves as a harmonic mean of Precision and Recall, balancing the trade-off between the two. It is especially beneficial when dealing with class imbalance, as it considers both false positives and false negatives.

Addressing reproducibility challenges in machine learning, a repeated 10-fold cross-validation (CV) strategy was implemented for the best EL model selection. The crash dataset was randomly split into training and testing samples, and five ensemble learners were fitted on the training dataset. A stratified 10-fold CV assessed model performances, with steps repeated ten times and scores recorded each iteration. The classifier with the highest average score was selected for further hyperparameter tuning to optimize performance.

### Model interpretation

The Shapley Additive exPlanations (SHAP) method [50] was used to interpret the optimal EL model, providing an extensive understanding of the significant variables derived from the RPLM. SHAP, a novel model interpretation technique introduced by Lundberg and Lee [50], is based on the Shapley value, a solution concept in game theory, originally proposed by Shapley [51]. The Shapley value for each variable is calculated as the average change in prediction when the particular variable is included, compared to when it is not, across all possible subsets of features. This concept is similar to the average marginal contribution of a variable.

In the context of the animal-vehicle crash (AVC) severity analysis, let *f(x)* represent our ensemble model that predicts the probability of a severe crash outcome given a set of crash characteristics *(x)* (including road, environmental, and vehicle factors). For each crash instance in the dataset, SHAP computes the contribution of each feature to the deviation from the average predicted probability of severe outcomes across the entire dataset. Mathematically, for a specific crash with features *(x)*, the prediction *f(x)* is decomposed as:


$$f(x)=\phi_{0}+\sum_{j=1}^{N}\phi j(x)$$ (7)

where $\varphi_{0}$ represents the base value (average prediction across the dataset), *N* is the total number of features, and $\varphi_{j}(x)$ is the SHAP value for feature *j*. The SHAP value is defined as:


$$\phi_{j}(f,x)=\sum_{S\subseteq N\backslash \{j\}s}\frac{|S|!(|N|-|S|-1)!}{|N|!}[f_{x}(S\cup \{j\})-f_{x}(S)]$$ (8)

where *N* is the set of all features, *S* is a subset of features excluding feature *j*, and *f*_*x*_*(S)* represents the expected model output when only the features in subset *S* are known.

The SHAP method was selected for its ability to provide both global and local interpretability, which is crucial for understanding the complex relationships in crash severity outcomes. Unlike other methods, such as LIME, which require repetitive model fitting, SHAP provides consistent analysis across the entire dataset with reduced computational complexity. Its additive property ensures that feature contributions are clear and intuitive, allowing policymakers to understand the findings.

The SHAP summary plot arranges predictors in descending order of significance towards the predicted severity outcome, illustrating each predictor’s local contribution. The spread of color density in the scatter plot indicates the significance, with colors representing predictor levels from low (blue) to high (red). The location of specific color from zero (either right or left) explains the significance of the level of that particular feature (either positive or negative) on the response variable. The SHAP feature dependency plots were used to reveal feature interactions and their joint effects on crash severity outcomes. For a pair of features *i* and *j*, the dependency effect is calculated as:


$${SHAP\;Dependency}_{i,j}(x)=\;\phi_{i}(x)\;conditioned\;on\;x_{j}$$ (9)

In the dependency plots, Shapley values are labelled on the y-axis and crash instances are distributed on the x-axis according to the primary feature levels. Crash instances are colored based on the levels of the secondary feature, which interacts most strongly with the primary feature. In SHAP dependency plots, the distribution of the color above the zero line indicates positive influence of the interacting feature levels on the response variable, which in this study is crash severity outcome.

## Results and discussions

### Estimation results of RPLM

Based on the best-fit model, 11 variables were identified as statistically significant. A summary of the estimation results for these 11 variables is provided in Table 3.

**Table 3:** Estimation results of RPLM model.

Variable Name [Reference Variable]	Estimate	Std. Error	z-value	Pr. (>|z|)
**Road Type: Freeway** [Ref.: Undivided]	1.313116	0.609641	2.154	0.0312 **
**Road Geometry: Horizontal Curve** [Ref.: Straight Road]	0.477491	0.289676	1.648	0.0992 *
**Road Surface Condition:** **Wet/ Sandy/ Cracks/ Others** [Ref.: Good]	0.73066	0.219526	3.328	0.0008 ***
**Weather: Dust/ Foggy/ Rainy/ Other** [Ref.: Clear]	1.477963	0.731362	2.021	0.0432 **
**Vacation: Yes** [Ref.: No]	0.303824	0.172679	1.759	0.0784 *
**Heavy Truck Involvement: Yes** [Ref.: No]	−1.18762	0.29469	−4.03	0.0000 ***
**Type of Animal: Donkey** [Ref.: Camel]	−1.0828	0.454295	−2.383	0.0171 **
**Side Fence Barrier: Need to replace** [Ref.: Good]	1.371049	0.653626	2.098	0.0359 **
**Speed Bumps: Good** [Ref.: Not Applicable]	0.59313	0.310584	1.910	0.0561 *
**Speed Bumps: Need to replace** [Ref.: Not Applicable]	1.06485	0.557031	1.912	0.0559 *
**Road with 3 lanes per Direction (Lane_3)** [Ref.: Lane_1]	−1.3549	0.553337	−2.449	0.0143 **
**Length of Road**	−0.00372	0.001647	−2.261	0.0237 **
**Road with 2 lanes per Direction (Lane_2)** [Ref.: Lane_1]	−0.32971	0.544495	−0.606	0.5448
**Standard Deviation of Lane_2**	3.806206	1.961919	1.94	0.0523 *

***, ** and * represent 99%, 95%, and 90% confidence interval, respectively.

Model Specifications:

Optimization of log-likelihood by BFGS maximization.

Log-likelihood: −916.57.

Akaike Information Criterion (AIC): 1909.1.

Bayesian Information Criterion (BIC): 2108.5.

Number of observations: 1403.

Number of iterations: 126.

Exit of maximum likelihood estimation (MLE): successful convergence.

Simulation based on 500 Halton draws.

The odds ratio results of RPLM show that severe animal-related road crashes are more likely to happen on freeways than undivided roads by a factor of 3.72 (e^1.313). Roads with three lanes per direction have 74% (100 × (0.258−1)) fewer chances of injuries and fatalities than one-lane roads. Roads with two lanes per direction (Lane_2) were found to be a random parameter with a mean of −0.32971 and a standard deviation of 3.806206. Using the cumulative distribution function of the normal distribution, it can be stated that for 47% of the observations, roads having two lanes per direction increase the probability of severe crash outcomes. The mean of Lane_2 not being statistically significant suggests that the unobserved heterogeneity of roads with two lanes in the sample varied from one location or event to another, thus cancelling positive and negative effects. This variation could be due to other road characteristics, such as traffic volume, or might relate to animal habitat or grazing points. Concerning road geometry, the odds of having severe animal-vehicle crashes are 61.20% higher on roads with horizontal curvature than on straight roads. Factors such as poor pavement, vacation periods, and adverse weather increase the likelihood of severe AVC outcomes. Good side fencing protection can decrease the chances of severe crash outcomes as the odds of having more injuries and fatalities are associated with damaged/ tampered/worn-out side fences that need replacement. As for the involvement of heavy trucks, the chances of severe outcomes are 69.51% less likely than the involvement of cars or buses. Collisions with camels are 2.95 times more likely to result in injuries and fatalities than those with donkeys. In addition to the interpretations based on odds ratio, further insights were sought regarding variable impacts on the crash severity outcome using ensemble machine learning approaches.

### Selection of best ensemble learner and interpretation of the results

A subset of the AVC dataset involving only the statistically significant 11 predictor variables was trained using the five EL classifiers to find out the best learner. The CatBoost classifier was found to have better overall accuracy and recall scores than others.

Table 4 summarizes the best performance metrics measured on the validation datasets. The hyperparameters of CatBoost classifier were tuned further to enhance its prediction performance resulting in an accuracy score of 0.61 and a recall score of 0.64.

**Table 4:** Model comparison of ensemble learners.

Ensemble Learner	CatBoost Classifier	Light Gradient Boosting Machine	Extreme Gradient Boosting	Random Forest Classifier	Extra Trees Classifier
**Accuracy**	0.61	0.58	0.57	0.59	0.58
**Recall**	0.63	0.61	0.61	0.58	0.56
**Precision**	0.61	0.59	0.60	0.60	0.59
**AUC**	0.63	0.62	0.62	0.62	0.61
**F1 Score**	0.62	0.59	0.60	0.59	0.57

**Fig 3:**
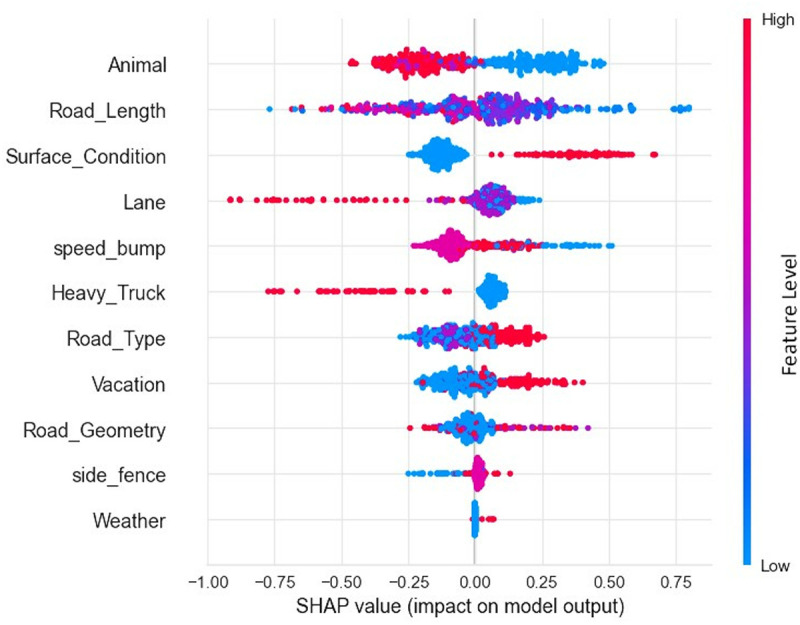
SHAP summary plot of statistically significant variables.

The findings of the CatBoost classifier were interpreted using SHAP plots. According to the SHAP summary plot shown in Fig 3, the type of animal involved has the greatest impact on the crash severity prediction, followed by the length of road and road surface condition. The distribution for each variable in the SHAP summary plot supports the findings of the estimation results of RPLM.

To interpret the results in an organized way, variables with similar attributes are discussed in the subsequent subsections utilizing both SHAP summary and dependency plots.

### Roadway features

**Fig 4:**
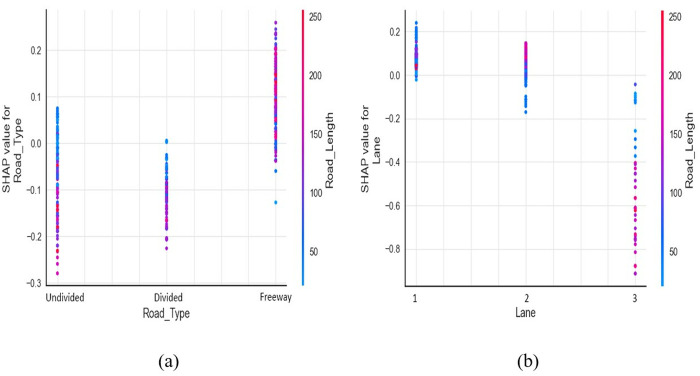
SHAP dependence plot: (a) road type & length of road, (b) number of lanes & length of road.

Fig 4a illustrates the impact of road type and road length on crash severity. For undivided roads, crashes tend to be more severe when the length is less than 70 kilometers. These shorter, undivided roads often have more conflict points, such as intersections, frequent stops, and turns, which could lead to a higher number of injuries and fatalities, particularly in rural areas. In contrast, animal-vehicle crashes on freeways are more likely to be severe, mostly on longer stretches of road (more than 130 kilometers). This observation aligns with the findings of Al-Bdairi, Behnood [21] and Erdogan [52]. Given their uninterrupted and access-controlled nature, vehicles on freeways or freeways are likely to travel at higher speeds. Combined with longer distances, this could lead to driver fatigue and drowsiness, potentially resulting in severe crashes, especially if animals suddenly appear on the road. A study by Ting, Hwang [53] suggests that on highways, the maximum safe limit to avoid fatigue-related crashes is 80 minutes of driving, which equates to a distance ranging from 133 to 187 kilometers in KSA, depending on the vehicle type. Divided multilane roads are expected to have fewer injuries and fatalities than the other two road types.

Fig 4b shows the interaction between the number of lanes and road length. Regardless of the road length, the chances of severe crashes are high on single-lane roads per direction, whereas the chances are low for roads with three lanes. With more lanes on rural roads, drivers are expected to get better space to avoid severe collisions with animals. Although unrelated to animal-vehicle collisions, similar conclusions were made by Se, Champahom [54] found less severe crashes in two-lane roads per direction compared to one-lane. Islam and Jones [43], while investigating pedestrian-at-fault crashes, also found that single-lane per direction roads have high probability of injuries than multilane roads in rural areas. Regarding roads with two lanes per direction, severe crashes are more commonly associated with longer roads. Conversely, shorter lengths may have varying severity outcomes, which could be influenced by unobserved circumstances.

**Fig 5:**
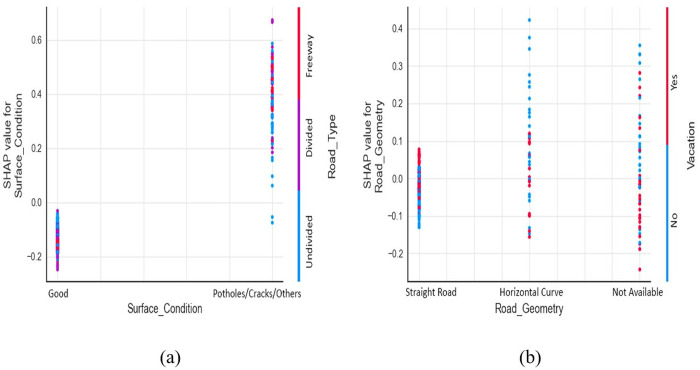
SHAP dependence plot: (a) surface condition & road type, (b) road geometry & vacation.

Pavement surfaces with distresses, potholes, or rutting are likely to have more severe crashes in all road types, as plotted in Fig 5a. This is likely due to the challenges in maneuvering vehicles on such poorly maintained road surfaces, especially on high-speed roads with less time to react [55].

According to Fig 5b, AVC on straight roads have more possibilities to result in injuries and fatalities when they occur on vacation, possibly due to over-speeding. The spread of blue dots with positive SHAP values in road sections with horizontal curvatures indicates that roads with curvatures are more sensitive to severe crashes, even without a rush for holiday trips. The scatter plot of road geometry in Fig 3 also suggests that AVC on horizontal curves are more vulnerable to severe crashes, possibly due to the limited visibility of the driver on the curves, leading to reduced reaction time. This finding is also evident in the work of Gharraie and Sacchi [23] and Savolainen and Ghosh [20].

### Road safety and maintenance items

**Fig 6:**
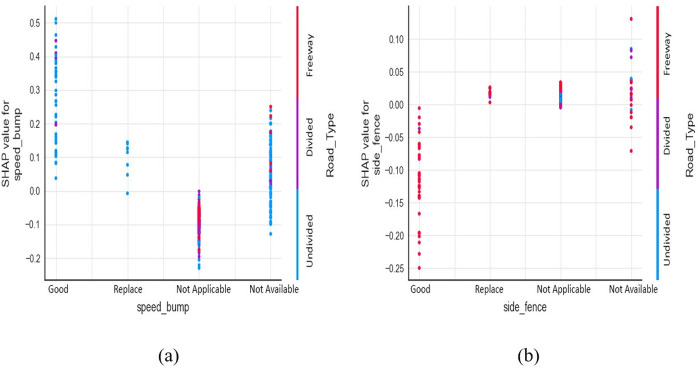
SHAP dependence plot: (a) speed bump & road type, (b) side fencing & road type.

Fig 6a shows the interaction effects of speed bumps and road type. The presence of speed bumps (whether in good or bad conditions) is likely to have a positive influence on severe outcomes of crashes occurring on divided and undivided roads. While speed bumps were associated with increased severity, this may reflect confounding due to road environment or driver behavior (e.g., speeding, limited visibility), rather than the speed bumps themselves. For instance, in rural areas where the speed limit is 80 kph or above, it is plausible that drivers, who may be distracted by mobile devices or impaired by limited night vision, could lose control of their vehicles upon encountering a sudden speed bump. This could lead to collisions with either animals, fixed objects, tripped rollovers, or rear-end crashes due to sudden vehicle braking.

Fig 6b shows the interaction effects of side fencing with road type. The scatter plot reveals a positive association of severe crashes in freeways when the side fencing is in a state of bad condition (either worn out or bent) which can lead to animal exposure on roads. The absence of side fences also increases the likelihood of injuries and fatalities on freeways and undivided rural roads. Although not related to severity, presence of well-designed and maintained side fencing alongside roads was found to be an effective way in reducing animal-vehicle collisions, as reviewed by Hedlund, Curtis [56].

### Environmental feature

Animal-vehicle collisions in adverse weather conditions are expected to have severe outcomes, as depicted in the SHAP summary plot (Fig 3). This finding is supported by the studies of Rahman, Das [26] and Gharraie and Sacchi [23]. Adverse weather conditions, such as rain or sandstorms, can create visibility hazards and reduce surface friction, increasing the likelihood of severe crashes [57]. However, the impact of these conditions on the model is relatively low compared to other variables. This may be due to the small proportion of crash occurrences (2%) in adverse weather conditions in the dataset.

### Other crash contributory variables

For animal type, Fig 3 suggests that collisions with camels (represented by blue spreads) have a positive influence on the severe crashes. Being heavier and taller compared to other animal types in KSA, the possibility of more injuries and fatalities from camel-vehicle collisions is expected.

Regarding vacation, the SHAP summary plot (Fig 3) indicates that crashes occurring during vacation periods (represented by red spreads) are more likely to result in severe outcomes. Almannaa, Zawad [58]) also found vacation to be one of the contributing parameters towards overall fatal crashes in KSA. During vacation periods, the likelihood of crashes increases, particularly in rural locations. These incidents often involve multiple vehicles, angular collisions, rear-end collisions, and instances where restraints are not used [59].

Concerning trucks, the negative dispersion of red dots on SHAP values in Fig 3 suggests that the involvement of heavy trucks contributes less to injuries and fatalities in crashes involving animals. This is consistent with the findings of Al-Bdairi, Behnood [21]. Since trucks are large in sizes and are capped to a relatively lower speed limit than passenger vehicles and buses, drivers may find it easier to control their vehicles in the event of a sudden animal appearance on the road the severity remains lower. However, the chances of fatality remains high for the animals if got hit by a heavy truck [60].

## Conclusions

Animal-vehicle crashes (AVC) are often overlooked in crash severity studies due to their relative infrequency. However, in regions like Saudi Arabia (KSA), where camels are a significant part of the animal population and often wander onto rural roads, the impact of AVC can be substantial. This research, a pioneering effort in KSA, examined the factors contributing to fatalities and injuries in AVC. The study analyzed 1,403 AVC on intercity and major intra-city roads, examining 24 variables related to roadway features, safety items, crash associated factors, and spatial, temporal, and environmental attributes.

A systematic approach was used to test the variables for multicollinearity and select potential variables for crash severity modeling using LASSO regularization. A random parameters logit model (RPLM) was employed to quantify statistically significant variables and account for unobserved heterogeneity in the dataset. A likelihood ratio test was conducted between a standard logit model and RPLM to justify the selection of the model. The estimation results of RPLM found one variable (roads with two lanes per direction) to be a random parameter and 11 statistically significant variables contributing to injuries and fatalities in animal-vehicle crashes. These variables were further examined using ensemble machine-learning techniques to seek more insights. Five ensemble learners were used to train the data and predict the crash outcome. The CatBoost classifier, chosen based on its superior average prediction scores, was tuned to improve accuracy. Shapley Additive exPlanations (SHAP) was utilized to interpret the findings of the tuned CatBoost classifier, providing detailed insights into the contributory factors’ relative significance and their interaction effects.

The findings of this study provide valuable insights for policymakers and road safety authorities to mitigate the severity of animal-vehicle crashes. Several practical implications emerge from the analysis. Roadside fencing along highways is critical for preventing animal intrusions from grazing areas and nearby localities. Implementing proper fencing combined with regular maintenance can significantly reduce AVC severity. Crashes occurring during vacations, which show a higher likelihood of severe outcomes, highlight the need for targeted interventions, including stricter enforcement of traffic regulations, enhanced traffic monitoring, and public awareness campaigns. Although heavy truck involvement was found to be less likely to contribute to severe crashes, the significance of truck involvement in crashes underscores the importance of increasing monitoring and inspection, particularly on roads lacking checkpoint stations. Similarly, periodic maintenance of road safety items, including speed bumps, is essential. According to the findings, poor pavement conditions also contribute significantly to severe AVC across all roadway types. Ensuring compliance with construction standards, incorporating accurate traffic forecasts during planning, and scheduling regular pavement maintenance are critical measures to address this issue. Uninterrupted freeway driving increases the risk of fatigue-related crashes, necessitating measures like frequent rest areas, fatigue-alert signage, and in-vehicle monitoring devices. Finally, the significant contribution of camels to AVC fatalities calls for safety awareness programs targeting camel owners to prevent animal exposure on roads. These actionable strategies can help reduce AVC severity and improve road safety.

## Limitations and future works

The research faced certain limitations due to some constraints. The roadway condition dataset was collected by a separate survey team at different times from the crash events. This temporal misalignment may introduce measurement error that could affect the precision of severity attribution. However, most infrastructure elements (e.g., pavement quality, signage, fencing) change slowly, especially on intercity roads. In addition, the use of LASSO regularization and robust standard errors in the RPLM helps mitigate potential bias from unobserved time-varying factors. Future studies are encouraged to employ real-time or near-real-time road monitoring systems to reduce such limitations. Additionally, due to data unavailability, factors such as traffic volume, road segment length, street lighting conditions, driver’s age, airbag deployment, seat belt usage, vertical curvature on roadway, and effect of vegetation or locality alongside rural roads could not be investigated. For future research, it is recommended to include these variables to provide a more comprehensive understanding of the factors influencing animal-involved crash severity. Exploring other methodologies, such as capturing unobserved heterogeneity using artificial neural networks can add new dimensions to animal-vehicle crash severity modeling.
